# Decreasing seagrass density negatively influences associated fauna

**DOI:** 10.7717/peerj.1053

**Published:** 2015-06-23

**Authors:** Rosemary M. McCloskey, Richard K.F. Unsworth

**Affiliations:** Seagrass Ecosystem Research Group, College of Science, Swansea University, UK

**Keywords:** Seagrass, Fish, Heterogeneity, Structural complexity, Habitat association, Structural complexity, Eelgrass, *Zostera*

## Abstract

Seagrass meadows globally are disappearing at a rapid rate with physical disturbances being one of the major drivers of this habitat loss. Disturbance of seagrass can lead to fragmentation, a reduction in shoot density, canopy height and coverage, and potentially permanent loss of habitat. Despite being such a widespread issue, knowledge of how such small scale change affects the spatial distribution and abundances of motile fauna remains limited. The present study investigated fish and macro faunal community response patterns to a range of habitat variables (shoot length, cover and density), including individual species habitat preferences within a disturbed and patchy intertidal seagrass meadow. Multivariate analysis showed a measurable effect of variable seagrass cover on the abundance and distribution of the fauna, with species specific preferences to both high and low seagrass cover seagrass. The faunal community composition varied significantly with increasing/decreasing cover. The faunal species composition of low cover seagrass was more similar to sandy control plots than to higher cover seagrass. Shannon Wiener Diversity (*H*′) and species richness was significantly higher in high cover seagrass than in low cover seagrass, indicating increasing habitat value as density increases. The results of this study underline how the impacts of small scale disturbances from factors such as anchor damage, boat moorings and intertidal vehicle use on seagrass meadows that reduce shoot density and cover can impact upon associated fauna. These impacts have negative consequences for the delivery of ecosystem services such as the provision of nursery habitat.

## Introduction

Many shallow marine habitats are structurally complex and contain an abundance of associated fauna. Such habitat therefore provides important functions for a diverse range of motile fauna including food provision, shelter from predation and providing opportune habitat for the growth and survival of young (Gillanders, 2006; Jackson et al., 2001).

Small scale habitat variation within marine habitats is commonplace (e.g., % cover, frond, branch or shoot density, canopy height) and can occur naturally due to fluctuations in the system, particularly as marine habitats are rarely homogenous in structure (Guidetti & Bussotti, 2000). Disturbances can accelerate and intensify habitat change, with continuous disturbance affecting the ability of habitats to recolonize and recover (Short & Wyllie-Echeverria, 1996). This results in the vegetation of many marine habitats becoming sparser and containing an increasing prevalence of halos.

Seagrass meadows are an example of a shallow water habitat where both natural (extreme weather events, over grazing and disease) and anthropogenic (nutrient overload, dredging and filling, pollution, destructive fishing, anchoring) disturbance can result in patchiness, variable cover and fragmentation (Hastings, Hesp & Kendrick, 1995; Short & Wyllie-Echeverria, 1996). When seagrass meadows occur in a shallow intertidal environment, the impacts of localised anthropogenic disturbance such as vehicle use, anchoring, bait digging and the presence of boat moorings can have significant impacts, resulting in patchy seagrass. What impact this small scale variability has on associated motile fauna has received limited examination, particularly with respect to the fish fauna.

The fish fauna present in seagrass meadows consists of a variety of permanent and temporary residents of all age ranges (Nakamura & Tsuchiya, 2008). The influence of environmental variation on the assemblages of fish is well documented with variations in community composition occurring on a daily (diel) (Unsworth et al., 2007), tidal (Unsworth, Bell & Smith, 2007), and seasonal basis (Garwood, Mulligan & Bjorkstedt, 2013). Diel variation may be attributed to species feeding preferences, with some immigration into meadows at night by active night feeders or species attempting to hide from predation (Robblee & Zieman, 1984; Unsworth et al., 2007), or decreases in the abundance of predators that rely on visual cues to catch prey (Clark, Ruiz & Hines, 2003). Less well understood is how fish communities vary as a function of space within seagrass meadows.

In order to investigate spatial change in seagrass fish communities theories of landscape ecology have been adapted and then applied for the study of seagrass ecology (Robbins & Bell, 1994). Subsequently a wide range of studies have been conducted which characterise landscape patterns in different ways. Although they use a variety of methodologies and terminologies, ultimately their aims are to investigate plant and animal interactions (reviewed in Bostrom, Jackson & Simenstad, 2006). Landscape scale studies (100s to 1,000s of metres) usually discuss fragmentation and connectivity issues based on ecological theory that stems from MacArthur and Wilson’s theory of island biogeography (MacArthur & Wilson, 1967), whereas within-patch/small scale studies contain some measure of complexity such as shoot density, canopy height, cover, leaf area or some combination of the above (Bostrom, Jackson & Simenstad, 2006). It is these small scale levels of seagrass variation and their consequences for fish that have received much less research effort and our understandings of the consequences of such small scale change are therefore limited.

It might be expected that as the seagrass increases in density so does the 3-dimentional complexity of the habitat. Studies in other habitats have shown that complex habitats contain higher species richness (Gratwicke & Speight, 2005), diversity (Webster, Rowden & Attrill, 1998) and abundance (Fredriksen et al., 2010).

Complex habitats contain more hide-spaces for prey and several studies show a non-linear relationship between seagrass density and predation success/intensity, where beyond a certain threshold of complexity predation success is significantly impacted (Reviewed in Heck & Orth, 2006). Complex habitats therefore provide suitable habitats for a variety of life, including vulnerable life stages such as juveniles seeking to avoid predation.

This paper aims to determine the response of the motile macro-fauna within a seagrass meadow to small-scale variability (e.g., habitat heterogeneity in terms of seagrass density). The research tests the null hypotheses that there is no link between the motile macro faunal assemblage and small scale habitat variability within a seagrass meadow, and that there are no individual species habitat preferences.

## Materials and Methods

Habitat assessment and seine hauls to sample the motile fauna were carried out in a disturbed and patchy intertidal *Zostera marina* seagrass meadow in Porthdinllaen, North Wales (see Plate 1) in August 2012 and June 2013 (52°56.600′N, 4°34.014′W). During 2012 a series of six seagrass plots over a gradient of varying habitat characteristics were quantified for their flora and motile fauna. Based upon the finding of the initial assessment a further study was conducted during 2013 to examine high density and low density seagrass.

### Habitat assessment

During the first sampling period six 6 × 6 m areas of seagrass were marked out and photographed using a high resolution digital camera (36 m^2^ was deemed suitable for fish assessment as the 8 m seine net could cover the sample area effectively during an initial trial). 36 × 1 m^2^ quadrat photos were taken to cover the entire plot. A 0.25 m^2^ gridded quadrat was thrown at random ten times within the plot in order to obtain an estimate of percentage cover and to measure shoot density and length. Percentage cover guides were used as a reference point (McKenzie, 2003). Shoot density was calculated by counting the number of shoots within 25 squares in each 0.25 m^2^ quadrat. Shoot length was measured by ignoring the top 20% of the shoots and taking up to five length measurements in each quadrat in order to obtain an average (McKenzie, Campbell & Roder, 2003). Study plots were initially chosen subjectively in order to reflect a range of apparent complexity that was later quantified in detail using multiple metrics. Complexity in the initial assessment represented increasing shoot density. In August 2013 a secondary, broader assessment of areas of low and high cover was carried out. Ten quadrats (6 m × 6 m area) were taken within visibly assessed low cover seagrass and ten quadrats were taken within high cover seagrass. Within each quadrat measurements of density, cover and shoot length were taken.

### Fish sampling

During August 2012 an 8 m beach seine net was hauled within six different predefined 36 m^2^ plots. The net was hauled through the plot (see Video S1) and the weighted ground line was lifted as it reached the shoreward edge of the plot, to avoid sampling within areas that had not been habitat assessed. Fish were identified and length measurements were taken. A total of 46 daytime beach seine hauls were carried out in the six different seagrass plots and 8 seine hauls in a sandy control sites. All seine netting was conducted on an incoming low tide. Three of the six plots were sampled one after each other as rapidly as possible on one tidal window, and the other three plots were sampled the following day. The sequential sampling order of the plots altered each sampling time.

During sampling in June 2013 the same fish sampling method was adopted but plots were not predefined, instead random sampling was carried out across the meadow within areas of low and high cover. These were quantified for their habitat characteristics after the fish assessment but initially picked based on broad estimates of high and low density. 8 seine hauls were carried out in low cover seagrass and 8 in high cover seagrass. All sampling was carried out within 1 h (±15 mins) after low water and in an average of 1 m water depth over the bed. Sampling sites were chosen so as to be accessible for seine netting on low water.

Sampling of juvenile (undersized) fish was conducted under a dispensation approval from the Provisions of Council Regulation 850/98 and The Marine and Coastal Access Act 2009. Dispensation was provided in the form of a letter from the Welsh Government fisheries office dated 10th April 2012.

### Data analysis

Data analysis was carried out using Primer v6 (Clarke & Gorley, 2006) and Sigma Plot v12.0. Fish abundance data was square root transformed to down-weigh the importance of high abundance species and a triangular resemblance matrix was created by analysing Bray Curtis similarity between the samples. Habitat variables was normalised and a resemblance matrix was created using Euclidean distance. The BEST (BIOENV) procedure was used to obtain the best correlations and sets of correlations between the resemblance matrices of the species abundance data and the habitat data, using Spearman’s rank correlations. Multi-dimensional scaling (MDS) plots were generated with super imposed Bray Curtis similarity clusters at the 40 and/or 50% similarity level in order to display the fish abundance data. ANOSIM (analysis of similarities) and pairwise tests were used to test the similarity between *a priori* defined groups of samples (i.e., plots, % cover level). SIMPER analysis (Similarity Percentages) was carried out in order to test for species contributions to the Bray Curtis similarity between and within *a priori* defined groups, again this method generates a permutations test and null distribution of the test statistic (Clarke, 1993). Shannon Wiener diversity was also assessed in Primer v6 and compared between high and low complexity samples using *t*-test in Sigma Plot v10. Spearman’s rank correlations were carried out in Sigma Plot to investigate the relationship between individual species and the habitat variables.

## Results

During a three week sampling period in August 2012, 19 species of fish, 2 species of Crustacean and 1 Cephalopod species were recorded in 48 seine hauls. The most abundant and frequently sampled fish species was the Sand Goby (*Pomatoschistus minutus*), which was found in 96% of the samples and averaged around 34 ± 27 individuals per seine net haul. Other abundant species were the Common Prawn (*Palaemon serratus*) and the Corkwing Wrasse (*Symphodus melops*).

During a one week sampling period in June 2013, 17 species of fish and 2 species of Crustacean were recorded. Common Prawn was the most abundantly sampled species followed by Pollack (*Pollachius pollachius*).

### Determining relevant habitat variables

Habitat variables from the six different 36 m^2^ plots are detailed in Table 1. BIOENV procedure showed that the variables that best described the patterns in the abundance data were components of small scale variation in cover of vegetation (average % cover, shoot density and shoot length). Average percentage cover alone gave the highest Spearman rank correlation coefficient (*ρ* = 0.588) at the 0.1% significance level, with no values generated by 999 permutations of the data labels equalling or exceeding the test statistic. The next highest correlation was achieved by combining three variables (*ρ* = 0.549), but this did not further elucidate the link between the habitat data and the fish assemblage patterns, and therefore percentage cover was used as the determinate variable in subsequent analysis.

**Table 1:** Seagrass flora metrics (average ± standard deviation) for the six 6 m × 6 m plots sampled during 2012 at Porthdinllaen, North Wales. Data collected using 0.25 m^2^ quadrats (shoot density, shoot length, percentage cover). A total plot percentage cover value was also included for the sample plot obtained from post processing of 36 × 1 m^2^ quadrat photos.

Plot	1	2	3	4	5	6
Seagrass % Cover %	16.2 ± 9.6	65.0 ± 22.4	16.1 ± 9.7	29.1 ± 40.6	27.4 ± 10.2	6.6 ± 6.4
Shoot density (per 0.25 m^2^)	49.6 ± 33.4	145.1 ± 39.9	64.0 ± 26.8	31.0 ± 38.1	54.8 ± 27.1	17.4 ± 11.8
Shoot length (cm)	18.8 ± 2.6	26.6 ± 4.1	17.3 ± 3.8	35.4 ± 14.0	21.6 ± 2.6	19.6 ± 1.1
Total cover % (per 36 m^2^)	63.0	91.0	78.0	43.0	30.0	12.0

### Fish and invertebrate community response to heterogeneity

**Figure 1:**
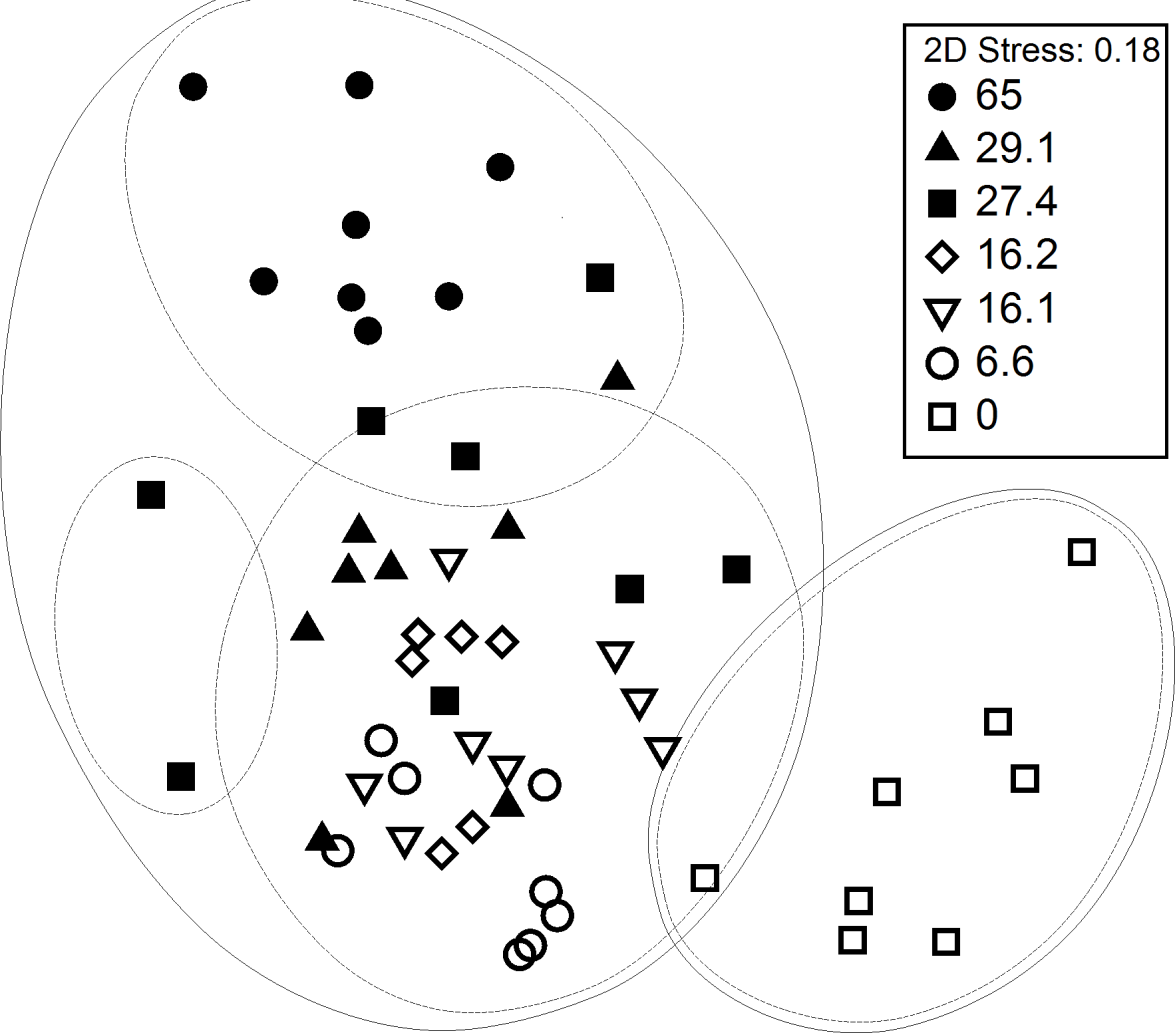
MDS ordination of fish and motile invertebrate assemblages at 6 plots of varying seagrass cover at Porth Dinllaen, North Wales. Ordination is superimposed Bray Curtis similarity clusters at the 40% (outer lines) and 50% (inner lines) level. Average percentage cover for each of the 6 plots is indicated by the key (Replicates within each % cover category are time series-replicates as the same plots were sampled over time).

Non-metric multidimensional scaling (nMDS) with superimposed Bray Curtis clusters at the 40% (outer lines) and 50% (inner lines) similarity level showed that fish and invertebrate sampled in seagrass meadows have different species assemblages patterns than those from the control (sand) (Fig. 1). Similarity clusters at the 50% level show that the 65% cover plot appears to be separated from the other seagrass samples and is similar at the 50% similarity level to a few samples of the next highest cover plots (29.1% and 27.4% cover). The rest of the samples within seagrass show little differentiation. The highest cover plot (65%), and the control plot (0%) and lowest cover plot (6.6%) show the most dissimilarity and therefore are positioned furthest apart on the plot.

The fish and invertebrate assemblages were found to vary significantly with respect to the different plots (*R* = 0.581, *P* < 0.001). Subsequent pairwise tests (Table 2) showed that all the seagrass plots are significantly different to the control (*p* < 0.005) with the highest cover plot (65%) showing the most dissimilarity (*R* = 1, *p* < 0.001). There is also some evidence of a similarity gradient between the highest and lowest cover plots i.e., the lower the cover of the plots, the more dissimilar they were to the 65% cover plot.

**Table 2:** *R* values generated by pairwise comparisons of the similarities in the fish and invertebrate assemblages between seagrass plots of varying floral density at Porthdinllaen, North Wales (ANOSIM). Global test statistic (*R* = 0.581) significant at the 0.1% level.

Cover (%)	65	29.1	27.4	16.2	16.1	6.6	C
**65**							
**29.1**	0.67						
**27.4**	0.44	0.38					
**16.2**	0.82	0.26	0.10				
**16.1**	0.90	0.34	0.39	0.19			
**6.6**	0.99	0.65	0.58	0.33	0.24		
**C (Sand)**	1.00	0.96	0.89	0.91	0.84	0.81	

### Species contributions

Percentage similarity contributions of the top 5 species causing similarity/dissimilarity between the plots were obtained using SIMPER (Table 3). Sand Goby showed some of the highest percentage similarity contributions overall and had higher contribution to low cover plots than high cover plots. Corkwing Wrasse, Common Prawn and Fifteen-Spined Stickleback (*Spinachia spinachia*) had the highest percentage contributions to the 65% cover plot and had no significant contributions to the 6.6% cover plot and the control. Four of the top five contributors made no significant contributions to the control samples.

**Table 3:** Percentage similarity contributions for the top five species causing the most similarity/dissimilarity between plots. Percentages obtained using Bray Curtis in SIMPER.

Similarity contribution (%)					
Cover (%)	Sand goby	Corkwing	Common prawn	Brown shrimp	Fifteen spined stickleback
65.0	10.48	28.52	40.56	0	12.49
29.1	53.14	17.1	15.14	0	4.25
27.4	46.58	6.78	22.52	11.37	7.72
16.2	65.45	8.42	15.09	8.15	0
16.1	59.84	18.68	0	7.02	0
6.6	72	0	0	11.65	0
0	72.9	0	0	0	0

### Assemblage response to high versus low percentage cover

**Figure 2:**
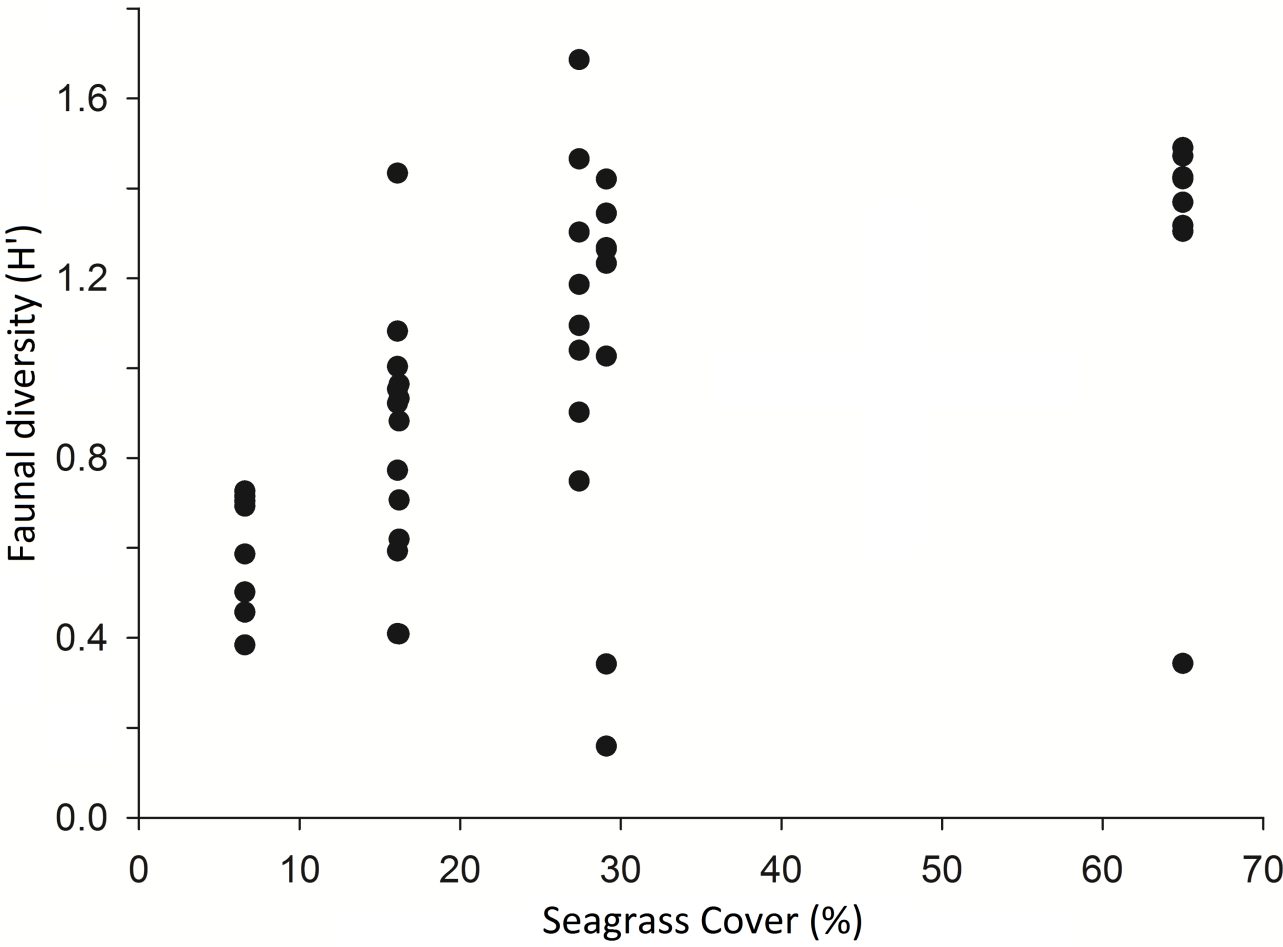
Diversity (Shannon Weiner) of taxa per seine net haul with respect to seagrass average percentage cover (per 0.25 m^2^) at Porthdinllaen, North Wales. Forty-six seine net samples (8 replicates within each % cover plot except plot 1 (16.2% cover) which had 6 replicates).

A comparison of 8 seine hauls in areas of low seagrass cover (2–30%) and 8 in areas of high seagrass cover (50–90%) showed that Shannon Wiener diversity (*H*′) was significantly higher in high cover samples (*H*′ = 1.35 ± 0.46) than in low cover samples (*H*′ = 0.83 ± 0.45) (*t*-test, *p* < 0.05) (see Fig. 2). Number of species present was also significantly higher in high (8.6 ± 2.4) versus low cover seine hauls (5.0 ± 1.3) (*t*-test, *p* = 0.002). There was no significant difference in the total number of individuals per seine haul (*t*-test, *p* > 0.05).

The species assemblages observed in high % cover seagrass were found to be significantly different to those in low % cover seagrass (*R* = 0.421, *P* < 0.001). SIMPER analysis showed that the Common Prawn, Pollack and Corkwing Wrasse were the highest similarity percentage contributors to the high cover samples, whereas Common Prawn, Pollack and Plaice (*Pleuronectes platessa*) were the highest contributors in the low complexity samples (Table 4). Eight different species combined contributed over 90% of the similarity in the high density samples whereas only three made up the cumulative % similarity contribution within the low density samples.

**Table 4:** Percentage similarity contributions for the top five species causing the most similarity/dissimilarity between high and low % cover seine hauls. Percentages obtained using Bray Curtis in SIMPER.

Similarity contribution (%)					
Cover (%)	Common prawn	Herring	Corkwing	Plaice	Pollack
High	34.02	5.95	11.54	0	23.96
Low	60.36	0	0	11.72	19.52

### Species responses to cover (%) variation

**Figure 3:**
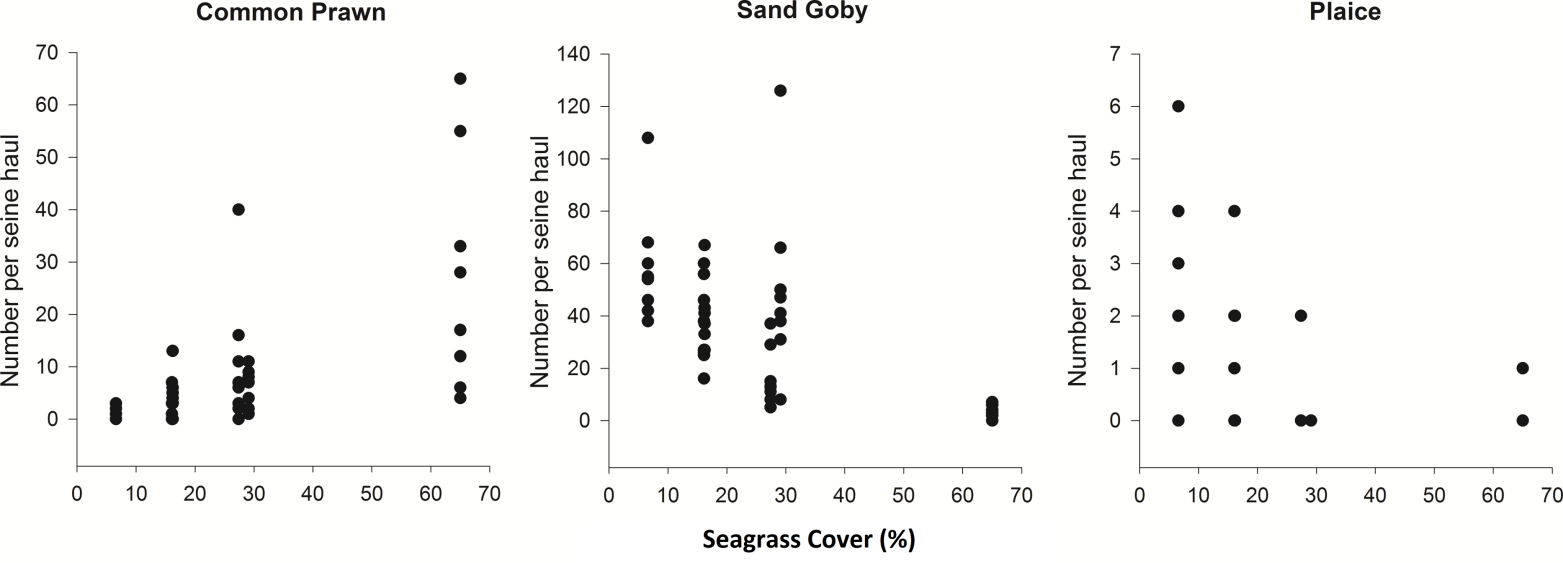
Density of common prawn, sand goby and plaice per seine net haul with respect to seagrass average percentage cover (per 0.25 m^2^) at Porthdinllaen, North Wales. Forty-six seine net samples (8 replicates within each % cover plot except plot 1 (16.2% cover) which had 6 replicates). Spearman’s rank correlation coefficients (A) *ρ* = 0.70, (B) *ρ* = − 0.60, (C) *ρ* = − 0.65 (*p* < 0.001).

Spearman’s rank correlations were carried out in order to investigate the relationship between the top 6 most abundant and frequently sampled species in the August 2012 samples and percentage cover. Common Prawn abundance had a strong positive correlation with percentage cover (*ρ* = 0.70, *p* < 0.001) and Sand Goby and Plaice abundance had negative correlations with increasing cover (*ρ* = − 0.60 and *ρ* = − 0.65 respectively, *p* < 0.001) (Fig. 3). From the other most abundant and frequently sampled species, Corkwing Wrasse showed a positive correlation with percentage cover (*ρ* = 0.57, *p* < 0.001), Brown Shrimp was negatively correlated (*ρ* = − 0.49, *p* < 0.001) and Fifteen-Spined Stickleback showed no relationship (*ρ* = 0.27, *p* > 0.05).

### Inter-annual variability

**Figure 4:**
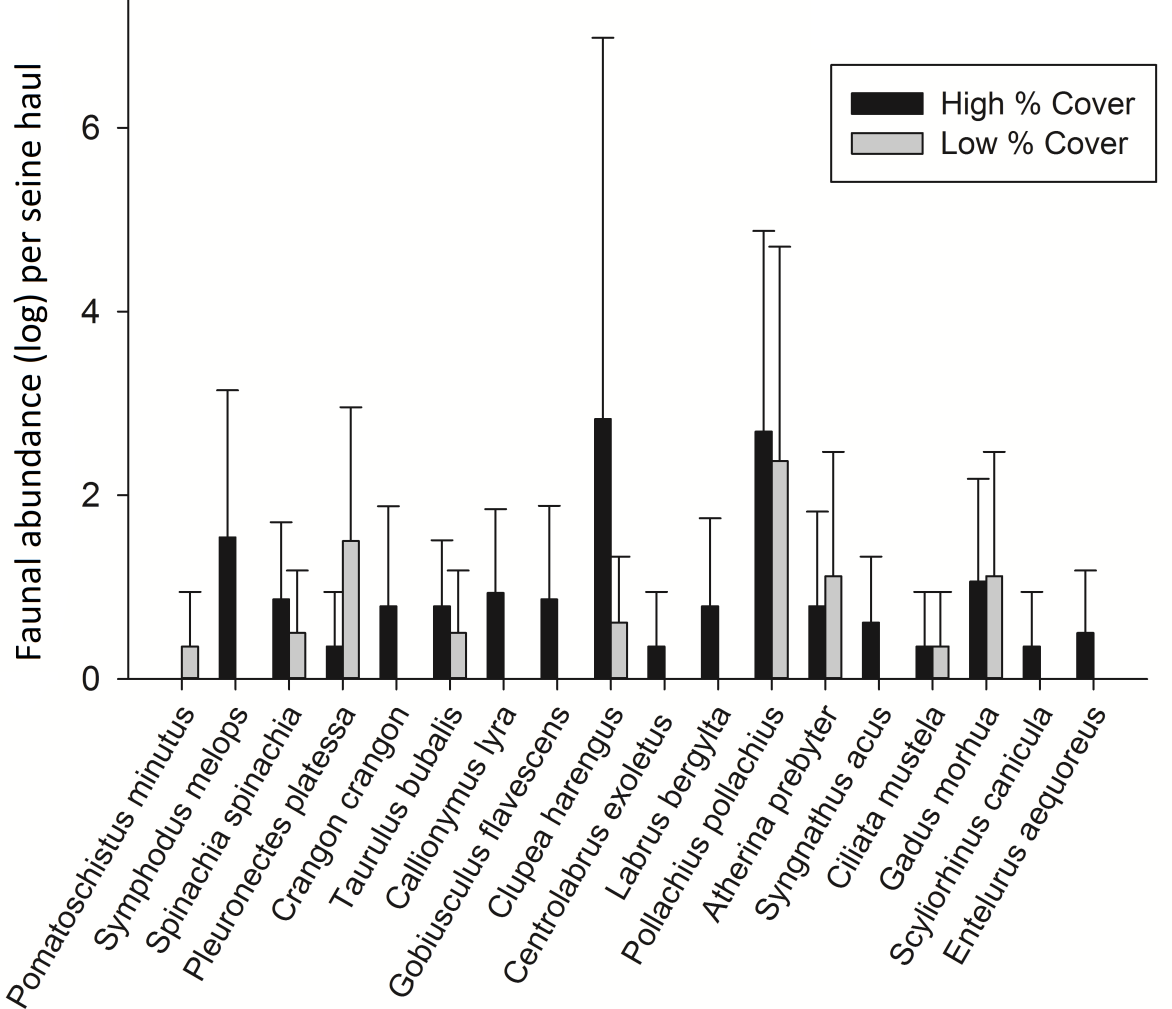
Mean fish abundance (± standard deviation) per seine net haul from 8 low percentage cover hauls and 8 high percentage cover hauls.

In order to test whether these species relationships were consistent, 8 high and 8 low cover seine net hauls were taken the following year. Some species changes were apparent from one year to the next. Most notable was the lack of Sand Goby, a species which had dominated samples the previous year. Pollack were also present in much higher abundance in 2013 and Atlantic cod were present in 56% of the 2013 samples but did not occur in any samples from 2012. Taking the common species from each sampling period, the theorised species relationships with percentage cover (Fig. 3) were tested by comparing high and low cover samples. Sand Gobies could not be further investigated due to their absence from the second season’s samples nor could Brown Shrimp due to their low numbers. There was no significant difference between the number of Common Prawn in high or low cover samples during 2013 (*t*-test *p* = 0.162). There was however a significant difference in the number of plaice in low cover (2.3 ± 2.1) versus high cover (0.13 ± 0.36) seine hauls during 2013 (Mann Whitney, *p* < 0.005) (Fig. 4). Corkwing Wrasses were significantly lower in abundance in low cover seagrass (no individuals sampled) than in high cover seagrass (2.4 ± 2.6 per seine haul). Other noteworthy differences between high and low cover samples were that three species of wrasse, 2 species of Pipefish, Dragonet, Two-Spotted Goby and Brown Shrimp were sampled exclusively in high cover seagrass.

## Discussion

The present study reveals strong evidence of the significant negative influence of small scale habitat variability on motile faunal assemblages, and consequently ecosystem service provision. We observed significantly higher faunal diversity and species richness in high versus low cover seagrass, with significant implications for the value of these habitats for the supply of ecosystem services (Cullen-Unsworth et al., 2014). Whilst there is extensive evidence of the impact of the decline in seagrass cover on associated benthic infauna (Attrill, Strong & Rowden, 2000; Bowden, Rowden & Attrill, 2001) there has previously been limited understanding of the influence of such small—scale variability on motile faunal assemblages. Such information is critical given the continuing disturbance of shallow water habitats such as seagrass and how this is increasingly fragmenting habitats (Short & Wyllie-Echeverria, 1996).

Changes in the overall community composition between sample plots of varying cover can be attributed within this study to the individual habitat preferences of select species. For example, there was a significantly higher number of Plaice found in low density compared to high density seagrass. Preference of bare over vegetated substratum has been documented in juvenile plaice (Wennhage & Pihl, 1994) and may be due to the need for suitable substratum to facilitate burial (Stoner & Ottmar, 2003) or as Plaice are mainly bottom feeders, dense seagrass may reduce their ability to locate prey successfully. Sand Goby were found in abundance in seagrass and sand, but were highest in abundance in the lowest cover seagrass.

These bottom dwelling Sand Gobies have been shown to prefer sand over mud substrate and predation of sand goby has been shown to increase when on the ‘wrong’ substrate (Tallmark & Evans, 1986). Wennhage & Pihl (2007) found that plaice occurred predominately in areas free of vegetation whilst sand goby preferred open sand flat habitat and they decreased in abundance with increasing vegetation. These species preferences are in agreement with the current study’s findings in seagrass habitats.

In the present study the patterns in the biota were best explained by small scale variation of within-patch metrics (within-patch average percentage cover, shoot length and shoot density). These variables were found to be significantly correlated. Despite the fact that there is some inter-correlation, average percentage cover, shoot density and shoot length all describe some aspect of within patch complexity. An increase in these metrics means an increase in the three-dimensional structure available for seagrass residents. Within-patch average percentage cover in this study effectively is acting as a proxy for shoot density and length. Various studies have attempted to avoid confounding and collinear variables in seagrass habitat studies by using artificial seagrass or experimentally manipulating plots (Gartner et al., 2013; Horinouchi & Sano, 1999). Most studies have used one seagrass ‘complexity’ measure (e.g., shoot density) and have avoided discussions of collinearity (Canion & Heck, 2009; Florido & Sanchez, 2010; Hughes et al., 2002).

Species–area relationships have rarely been applied and investigated in marine environments, and a review by Bostrom, Jackson & Simenstad (2006) on the effects of seagrass landscapes on their associated fauna found that no clear patterns emerged from studies which examined patch size effects. Hirst & Attrill (2008) found that the size of *Zostera* patches made no impact on diversity; however the presence or absence of seagrass made a significant difference. Hovel & Lipcius (2001) also found that survival of Blue Crab (*Callinectes sapidus*) increased with habitat complexity regardless of patch size. Although it is suggested by some that a hierarchical landscape approach is needed when investigating animal-habitat interactions in the marine environment (Pittman, McAlpine & Pittman, 2004), the current study found significant responses of motile fauna to small scale complexity variation and individual species habitat preferences which resulted in changes in the community structure in response to a gradient of habitat change at the patch scale. Regardless of scaling issues, these small scale patterns can be translated to a larger scale and provide information about the potential effects of degradation.

**Figure 5:**
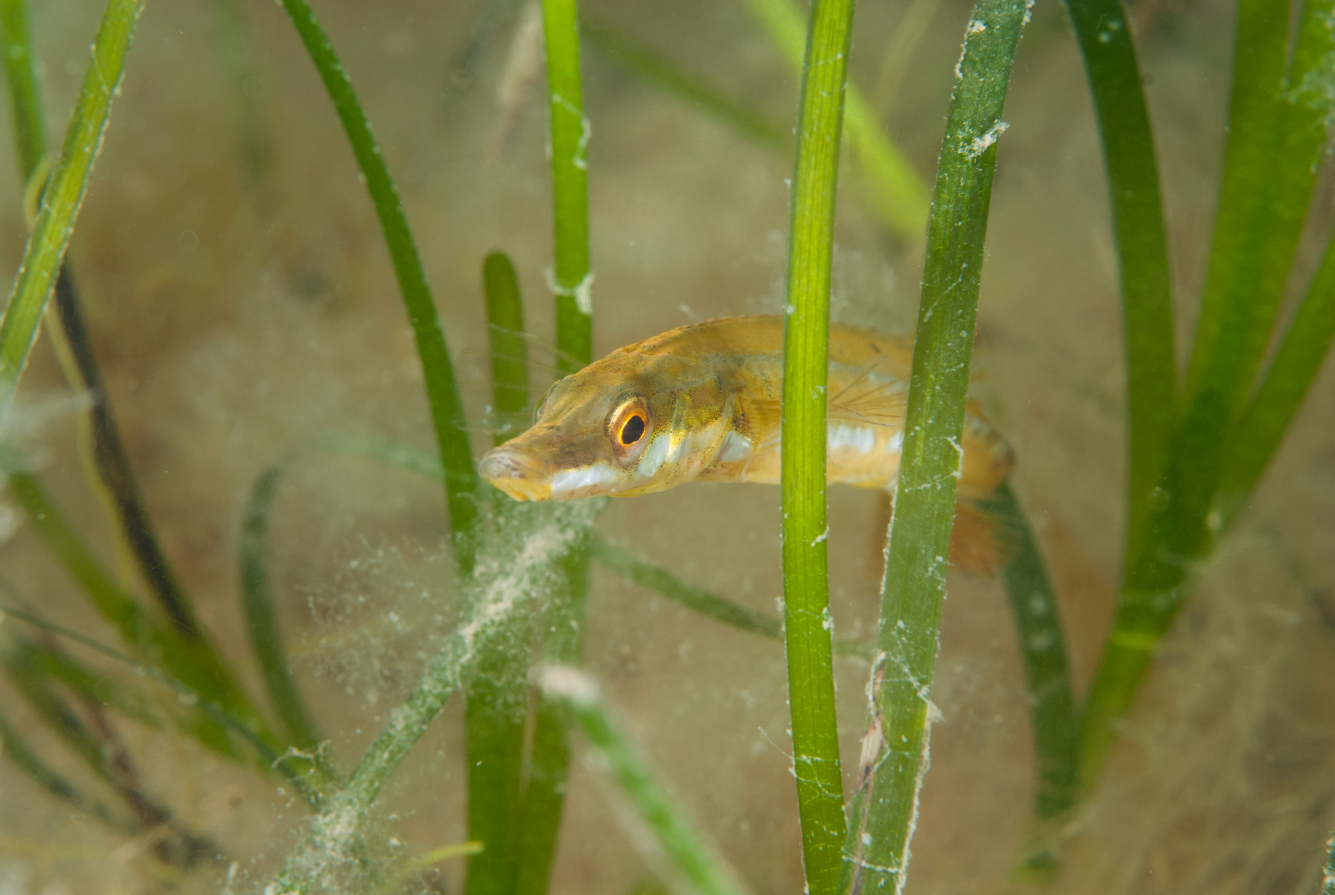
Seagrass (*Zostera* marina) at Porthdinllaen, North Wales. Seagrass at Porthdinllaen provides important habitat for many species of fish and invertebrate, including the Fifteen spined stickleback (*Spinachia spinachia*) (Photo credit: Frogfish Photography).

Finally the Shannon-Wiener diversity (*H*′) of seagrass plots was positively correlated with increasing within-patch cover and the average diversity from high cover plots was significantly greater than that of low cover plots. Several studies have had similar findings about the diversity promoting function of higher cover/density seagrass (Hughes et al., 2002; Webster, Rowden & Attrill, 1998). The association of various species with complex habitats may vary due to predator–prey interactions. As previously discussed dominant conspecifics or competing species may exclude others from preferential habitat (Coen, Heck & Abele, 1981; Lammers, Warburton & Cribb, 2009). The presence of a predator can also modify habitat selection (Sogard & Olla, 1993). In a laboratory study Tait & Hovel (2012) demonstrated a clear behavioural choice in juvenile Giant Kelpfish, which displayed preference for high complexity artificial seagrass, regardless of either predator risk or food availability. In conclusion the spatial configuration of species within seagrass meadows can be a function of individual species habitat preferences which are influenced by predator–prey interactions, competition for space and behavioural choices. High complexity habitats are the preferred habitat of a greater diversity of species than low complexity habitats (Fig. 5). Disturbances and habitat destruction can affect the ecosystem functioning of meadows, causing trophic cascades and an un-balancing of the system (Duffy, 2006). Severe disturbance results in species turnover and shifts in seagrass fish assemblages which result in a loss of diversity and biomass (Pihl et al., 2006).

Sizes of fish sampled within the meadow indicate that the majority of commercial fish sampled were below the age of sexual maturity, supporting the premise that these meadows have a high ecosystem service value as nursery habitats. Important commercial fish species found as juveniles included the Herring (*Clupea harengus*), Pollack (*Pollachius pollachius*), Plaice (*Pleuronectes platessa*), Dab (*Limanda limanda*), Atlantic Cod (*Gadus morhua*), and European Eel (*Anguilla anguilla*). On average commercial species contributed to only 2.2% of the total number of individuals per seine haul in 2012, but contributed to 26.9% in 2013. This was mainly due to the increased abundance of Pollack and Atlantic Cod in 2013. Other UK and Northern European seagrass studies have found high abundances of juvenile Pollack, Plaice and Herring and Atlantic Cod in seagrass (Bertelli & Unsworth, 2014; Lilley & Unsworth, 2014; Peters et al., 2014). The present study found that the abundances of these commercial species were variable over time and the abundance and distribution of Plaice was dependent on habitat type.

## Conclusions

In conclusion we find that seagrass meadows with high structural complexity are more valuable habitats for a broader range of motile fish and invertebrate fauna than low complexity meadows. These highly complex seagrasses consequently have a higher ecosystem service value, particularly as the species sampled were mostly juveniles of commercial importance to fisheries. Variable species distribution within and between habitats of varying complexity may be attributed to factors such as individual species habitat preferences (behavioural), predator–prey interactions and inter and intra specific competition. Seagrass meadows are increasingly subjected to the impacts of disturbance from small scale factors such as boat anchoring, the use of static moorings and the exploitation of associated fauna. Present research suggests that these activities if damaging the seagrass will have an impact upon associated fauna, many species of which are of commercial fisheries importance.

## Supplemental Information

10.7717/peerj.1053/supp-1 Appendix S1 Raw data as supplementary file Raw data (fish catches) collected at Porthdinllaen during 2012 and 2013 using a seine net.

10.7717/peerj.1053/supp-2 Video S1 Seine netting for fish and macro fauna at Porthdinllaen, North Wales

## Additional Information and Declarations

### Field Study Permissions

The following information was supplied relating to field study approvals (i.e., approving body and any reference numbers): Approval was provided in the form of a letter from the Welsh Government fisheries office dated 10th April 2012. This gave the authors dispensation from the PROVISIONS OF COUNCIL REGULATION 850/98 and THE MARINE AND COASTAL ACCESS ACT 2009.
